# Remote work burnout, professional job stress, and employee emotional exhaustion during the COVID-19 pandemic

**DOI:** 10.3389/fpsyg.2023.1193854

**Published:** 2023-06-01

**Authors:** Alina Costin, Alina Felicia Roman, Raluca-Stefania Balica

**Affiliations:** ^1^Center of Research Development and Innovation in Psychology, Faculty of Educational Sciences Psychology and Social Work, Aurel Vlaicu University of Arad, Arad, Romania; ^2^Department of Education and Communication Sciences, University of Craiova, Craiova, Romania

**Keywords:** remote work burnout, professional job stress, employee emotional exhaustion, COVID-19, pandemic

## Abstract

Many studies have investigated how organizational support systems, remote work adaptation, and control over scheduling reduced psychological burnout and occupational stress, thus improving employee wellbeing during the COVID-19 pandemic. This systematic literature review has analyzed significant published peer-reviewed evidence concerning how remote employees lacking constant organizational support during the COVID-19 outbreak experienced escalated job demands, professional strain, low satisfaction and performance, and increased burnout. Throughout February 2023, a quantitative literature review covering scholarly databases such as the Web of Science, Scopus, and ProQuest was performed, with the following search terms: “COVID-19” + “remote work burnout,” “COVID-19” + “professional job stress,” and “COVID-19” + “employee emotional exhaustion.” By inspecting research published between 2020 and 2022, a total of 311 articles satisfied the eligibility criteria. Excluding sources in PRISMA terms, 44 empirical sources were finally selected. Methodological quality assessment tools such as Assessing the Methodological Quality of Systematic Reviews (AMSTAR), Appraisal tool for Cross-Sectional Studies (AXIS), Mixed Methods Appraisal Tool (MMAT), and Systematic Review Data Repository (SRDR) were employed. Data visualization tools (VOSviewer and Dimensions), integrating layout algorithms and bibliometric mapping, were harnessed. The scope of this study does not include how taking breaks and time management in a psychologically safe environment prevented remote work burnout and increased productivity during the COVID-19 pandemic. Subsequent analyses should be developed on how remote work time and stress management—by using burnout assessment tools—will result in coherent workplace behaviors and processes, meeting organizational expectations and reducing emotional stress and workplace pressure.

## Introduction

Difficulties in handling remote work or difficulties in carrying out professional tasks effectively are associated with burnout and emotional stress. Isolated and unsupported employees experienced professional detachment and inefficacy, workplace disengagement, escalated job-related demands, employee burnout, and emotional exhaustion (Dionisi et al., 2021; Holmes et al., 2021; Rapp et al., 2021; Singh et al., 2022) during the COVID-19 pandemic. Remote workers typically experienced low levels of positive feelings, triggering unpleasant emotions, and developed burnout syndrome and an acute response to professional stress. Enforced remote work shaped employee performance in terms of reduced productivity, work engagement, and job satisfaction, decreasing subjective and psychological wellbeing.

Workplace social support is needed for technology-mediated remote work to improve professional engagement and the psychological wellbeing of employees. Incoherent human resource management decisions, lack of supportive working environment, lack of social connectedness, and diminished job autonomy, satisfaction, and performance decreased cognitive performance (Crippa et al., 2021; Gemine et al., 2021; Oksanen et al., 2021a; Spagnoli et al., 2021; Zhang et al., 2021), while emotional and psychological distress, occupational burnout syndrome, chronic workplace stress, and turnover intentions intensified during the COVID-19 pandemic. Low work morale and engagement resulted in a decrease in employee psychological health and efficacy. Working from home led to a feeling of being undervalued and not being trusted enough to undertake specific tasks among employees.

## Methodology

**Figure 1:**
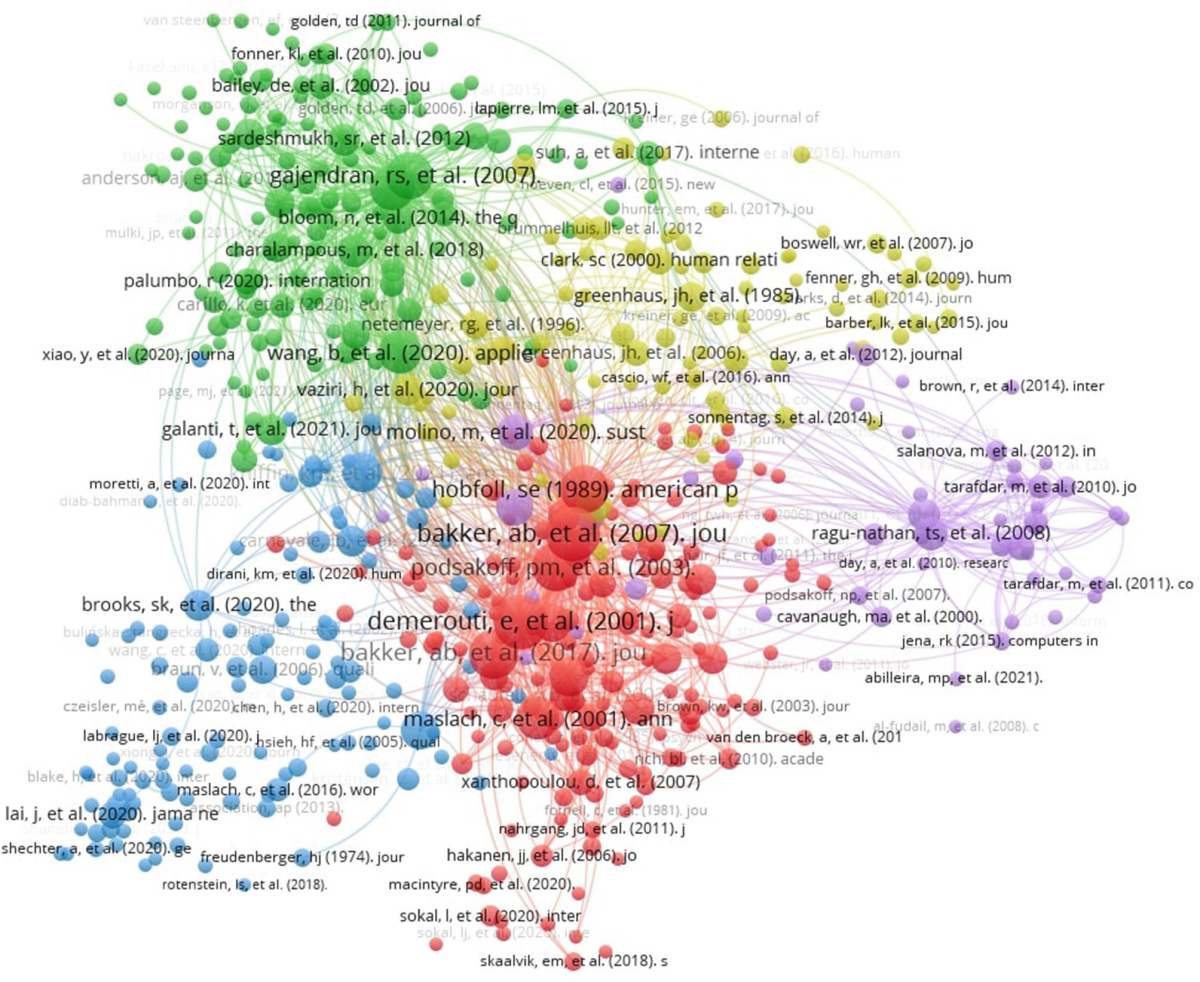
Cocitation covering the topic.

Throughout February 2023, a quantitative literature review covering scholarly databases such as the Web of Science, Scopus, and ProQuest was performed, with the following search terms: “COVID-19” + “remote work burnout,” “COVID-19” + “professional job stress,” and “COVID-19” + “employee emotional exhaustion.” By inspecting research published between 2020 and 2022, a total of 311 articles satisfied the eligibility criteria. Excluding sources in PRISMA terms, 44 empirical sources were finally selected (Table 1). Methodological quality assessment tools such as Assessing the Methodological Quality of Systematic Reviews (AMSTAR), Appraisal tool for Cross-Sectional Studies (AXIS), Mixed Methods Appraisal Tool (MMAT), and Systematic Review Data Repository (SRDR) were employed. Figure 1, covering cocitation, represents data visualization tools (VOSviewer and Dimensions) integrating layout algorithms and bibliometric mapping.

**Table 1:** Topics and types of identified and selected scientific products.

**Topic**	**Identified**	**Selected**
COVID-19 + remote work burnout	108	16
COVID-19 + professional job stress	102	14
COVID-19 + employee emotional exhaustion	101	14
**Type of paper**		
Original research	276	44
Review	5	0
Conference proceedings	11	0
Book	2	0
Editorial	17	0

Source: Processed by the authors. Some topics overlap.

## Remote work burnout during the COVID-19 pandemic

Throughout enforced remote work scenario during the COVID-19 crisis, technology exhaustion increased and subjective wellbeing diminished, contributing to job burnout, anxiety, and fatigue (Bakken and Winn, 2021; Jimenez-Gomez et al., 2021; Allgood et al., 2022; Singh et al., 2022), thus impacting employee performance. Working remotely caused feelings of being less connected with the organization and colleagues, thus configuring social isolation, enabled being engaged in decision-making fostered feelings of control and reduced burnout. Remote work intensified tensions of work–life balance among employees during the COVID-19 pandemic, but such conflicts could be alleviated by instrumental leadership and by a sense of social belonging, resulting in lower levels of burnout. Supervisor social support, constant and relevant information exchange across organizational teams, and planning on time management reduced burnout and improved productivity in remote workers.

Taking time off and managing mental health and workload efficiently can increase productivity and prevent remote work burnout. Work–home interference can suppress job engagement and satisfaction while amplifying professional burnout and turnover intentions (Chi et al., 2021; Mahmoud et al., 2021; Michel et al., 2021; Ninaus et al., 2021) due to altered organizational demands and resources. Cognitive and psychological strains associated with job insecurity and increased workload configured job-related burnout during the COVID-19 crisis. Demanding working conditions and lack of workplace flexibility affected employee job satisfaction and psychological wellbeing, leading to low work performance, turnover intentions, and physical and cognitive burnout. Occupational burnout can worsen not only job satisfaction but also the perceptions of predicted employee job insecurity and unsatisfactory organizational outcomes, in relation to COVID-19.

Emotional exhaustion and job burnout can be mitigated by remote work environment and organizational factors (Kniffin et al., 2021; Liberati et al., 2021; Spagnoli et al., 2021; Trombello et al., 2022) with regard to workload management. COVID-19-related organizational changes led to enhanced team creativity and innovation; to operational looseness and autonomy; to social isolation and loneliness; and to employee job burnout. Working remotely for a long time caused employee burnout, emotional exhaustion, psychological strain, reduced job performance, high turnover, and low levels of professional accomplishment. Additional tasks performed by remote staff exacerbated feelings of psychological distress, social helplessness, professional isolation, turnover intentions, occupational stress, and job burnout.

Feeling exhausted due to insufficient rest while working remotely can result not only in long-term mental health problems but also in lower productivity. Job burnout, remote work stress, and increased workload (Dionisi et al., 2021; Rapp et al., 2021; Upadyaya et al., 2021) affected COVID-19-related professional engagement and occupational wellbeing. Perceived stress and burnout affected employee mental wellbeing, thus reducing professional efficacy. Remote workers developed burnout syndrome and experienced job stress. An intensified prevalence of boundary violations and undesired professional disruptions during the COVID-19-related demanding work environment amplified job stress and burnout, negatively affecting work engagement and occupational identity.

Employee burnout and chronic job stress associated with long working hours can negatively affect work quality and personal relationships, consequently impacting mental health and psychological wellbeing. Attitudes, perceptions, and experiences associated with remote work (Crippa et al., 2021; Oksanen et al., 2021b; Van Zoonen et al., 2021; Zhang et al., 2021) negatively impact work–life balance, resulting in escalated prevalence of emotional exhaustion and burnout symptoms. Perceived remote work stressors and sudden organizational changes intensified psychological burnout and strain, leading to emotional exhaustion during the COVID-19 crisis. Sociodemographic differences, in addition to personality traits, articulated the remote work climate, bringing about psychological distress, increased mental health risks, and occupational burnout.

## Professional job stress during the COVID-19 pandemic

Remote work-related technostress and psychological ill-health (Hayes et al., 2021; Miguel-Puga et al., 2021; Trinidad, 2021; Singh et al., 2022) reduced productivity, engagement, and job satisfaction during the COVID-19 crisis. Persistent burnout due to increased workload influenced sleep disruption, acute stress, state anxiety, and derealization symptoms, negatively affecting the work–life balance. Technology-facilitated remote working resulted in higher levels of work-related stress and burnout due to COVID-19 restrictions, bringing about low work productivity. Organizational supports, together with decision engagement, are related to increased job satisfaction and social–emotional wellbeing, decreased burnout, and the likelihood of leaving the company for remote workers not experiencing heightened stress or having low pay.

Technology-mediated remote work led to work exhaustion, fatigue, psychological stress, burnout, and work–life balance conflict (Chi et al., 2021; Oksanen et al., 2021a; Shockley et al., 2021; Vargas Rubilar and Oros, 2021), which challenges private and professional life boundaries, thus affecting employees with poor technological skills and poor supervision and guidance throughout the COVID-19 crisis, while compromising job-related resources. Employee burnout and turnover, emotional and physical wellbeing, and work stress affected work–life balance and led to adverse job outcomes. Inadequate remote working conditions, work overload, and prolonged professional efforts to meet organizational demands brought about perceived occupational stress, psychophysical symptoms, and job burnout. Communication quality, expectation-setting processes, and socially supportive information exchanges were negatively associated with heightened job burnout and organizational stress and positively associated with work-related demands, professional performance, and psychological wellbeing.

The COVID-19 pandemic-related job stressors and psychological symptoms were related to professional burnout, emotional exhaustion, physical fatigue, and cognitive weariness (Ingusci et al., 2021; Michel et al., 2021; Ninaus et al., 2021; Salvesen and Berg, 2021), negatively impacting employee psychological wellbeing. Collaborative remote workplaces and coherent organizational resources optimized work engagement and job performance, decreasing cognitive and emotional demands, behavioral stress, time pressure, and professional requirements. Stress-related mental health issues and perceived levels of physical and cognitive burnout affected job satisfaction and the work–life balance. Remote workers struggled with occupational burnout, daily job stressors, emotional labor, and work–life balance issues.

Flexible work schedules together with positive work environments, constant virtual social interactions with the other staff members, and remote working capabilities mitigated burnout and improved job satisfaction and work–life balance (Ayyala et al., 2021; Liberati et al., 2021; Shipman et al., 2021; Upadyaya et al., 2021), thus reducing COVID-19-related stress, anxiety, and professional isolation. The COVID-19 pandemic intensified employee stress and job burnout, resulting in perceived low productivity and remote workplace disturbances. Reduced social contacts and loneliness associated with job demands and resources brought about stress and anxiety. Unsettling alterations, stressful incidents, and situational strains concerning working practices, social support, and professional support under severe pressure and time constraints due to remote–access technologies sometimes led to staff turnover.

Flexible jobs, time management, and emotional team support can improve employee health, productivity, performance, and creativity, reducing workplace stress, anxiety, burnout, and isolation. In the COVID-19 context, increased workload pressures and demanding remote working conditions acting as emotional and psychological burdens (Holmes et al., 2021; Liberati et al., 2021; Van Zoonen et al., 2021) involved prolonged psychological stress, emotional exhaustion, and low professional accomplishments. Organizational support decreased workplace stress, which brought about feelings of hopelessness. Work stressors in terms of high workload and job insecurity amplified work–life conflict, intensifying psychological strain and decreasing perceptions of social support.

## Employee emotional exhaustion during the COVID-19 pandemic

Workplace stress, strain, and uncertainty, employee psychological wellbeing and fatigue, increased cognitive strain and social isolation, emotional exhaustion, and decreased job satisfaction (Allgood et al., 2022; Bakarich et al., 2022; Mosleh et al., 2022; Selvaskandan et al., 2022) elevate occupational burnout and work–family conflict. COVID-19 working arrangements remotely led to feelings of depersonalization, emotional exhaustion, role stress, and overload, reduced personal accomplishment, and job burnout, increasing turnover intentions. Excessive work overload had, as a consequence, staff stress, emotional draining, professional burnout, and employee turnover. COVID-19-related remote working patterns, increased workloads, and living arrangements influenced the work–life balance negatively, giving rise to reduced job satisfaction, persisting job fatigue and burnout, organizational workload, decreased productivity, emotional exhaustion, workforce attrition, and low professional accomplishments.

Overwhelmed and emotionally drained remote team members due to long working hours experience mental strain signs and need asynchronous workflows, in addition to taking breaks and time away from organizational tasks. Elevated emotional and mental demands due to lack of organizational support (Bakken and Winn, 2021; Hayes et al., 2021; Meynaar et al., 2021; Miguel-Puga et al., 2021) decreased employee quality of life and work productivity during the COVID-19 pandemic. Burnout and emotional exhaustion are negatively related to work engagement and organizational resilience. The COVID-19-related psychological distress and emotional exhaustion were associated with depression, anxiety, and depersonalization symptoms among remote workers. Reduced social interactions and excessive workload negatively affected social and psychological health, leading to emotional exhaustion and increased burnout.

Remote workers were severely affected during the COVID-19 crisis (Gemine et al., 2021; Ingusci et al., 2021; Jimenez-Gomez et al., 2021; Rapp et al., 2021) and experienced distractions throughout work hours, decreased productivity, emotional exhaustion, job insecurity, work-related stress, and intensified burnout. Physical and emotional load, psychological fatigue, emotional pressures, and excessive workloads brought about increased professional burnout. Demands and resources had to be balanced across organizations to diminish emotional exhaustion, professional discomfort, and job burnout while optimizing motivational processes, work performance, and psychological wellbeing. Mental and emotional stress related to increased job demands and workload reduced employee motivation and diminished workplace wellbeing.

Decreased employee psychological health (e.g., emotional and physical draining) associated with job instability and loss, lack of organizational support, and professional turnover intentions (Chen and Eyoun, 2021; Holmes et al., 2021; Morse and Dell, 2021; Nabe-Nielsen et al., 2022) are negatively associated with perceptions of personal accomplishment, organizational commitment, and job performance. Remote employees experience emotional distress due to poor organizational support. The COVID-19-related emotional distress negatively influenced organizational communication, psychological wellbeing, and work–life balance. Worries related to the volume of job tasks and managing the working conditions during remote work increased adverse emotional reactions, unsatisfactory mental health, and perceived burnout and occupational stress.

## Discussion

Adverse behavioral and emotional feelings (Lăzăroiu et al., 2021a,b) and psychological distress (Ciobanu et al., 2019) increased employee burnout. Prolonged COVID-19-related employee burnout fatigue and emotional exhaustion associated with working from home (Chen and Eyoun, 2021; Hayes et al., 2021; Ninaus et al., 2021; Mosleh et al., 2022) required mental health support. Due to the COVID-19 pandemic-related restrictions (Lăzăroiu et al., 2020) and lack of organizational support, individuals working remotely experienced intensified perceived stress and job burnout, in addition to physical and emotional exhaustion. Increased occupational burnout levels decreased employee job satisfaction, negatively affecting the work–family balance.

Remote working diminished workplace and social connections (Chen and Eyoun, 2021; Krug et al., 2021; Shipman et al., 2021; Shockley et al., 2021) and negatively influenced the mental health (Sheares et al., 2020) and psychological wellbeing of employees during the COVID-19 pandemic. Resourceful and resilient organizations (Privara, 2022) operating remotely improved mental wellbeing and job satisfaction (Nemţeanu and Dabija, 2023), further optimizing employee financial security and reducing layoffs. The COVID-19-related emotional exhaustion and job insecurity (Popescu Ljungholm and Olah, 2020) affected perceived organizational support (Nemţeanu et al., 2022) and employee mindfulness in terms of human resource management practices. The remote work experiences in terms of cognitive and affective processes (Robinson et al., 2021) influenced job performance and employee wellbeing, thereby persuading organizational behavior.

Counterproductive behaviors (Lăzăroiu and Adams, 2020) while performing remote work can lead to employee burnout, professional dissatisfaction (Mikołajczak, 2021), and job turnover intentions. The COVID-19-related remote work was associated with physical and emotional exhaustion (Bratu, 2020a,b), intensified burnout risk prevalence, diminished personal accomplishment (Landmesser, 2021), decreased employee involvement (Nemţeanu et al., 2021a,b), low-performance symptoms, and generalized fatigue (Bruyneel et al., 2021; Milch et al., 2021; Venkatesh et al., 2021) brought about by increased workload. Growing workload (Liu et al., 2021), emotional and chronic workplace stress, increased psychological distress, low personal accomplishment (Chang and Wu, 2021), and decreased job satisfaction were associated with burnout.

Anxiety, depression, emotional exhaustion, feeling less valued, inefficient social support networks (Davis, 2021), and work overload (Bruyneel et al., 2021; Prasad et al., 2021; Van Zoonen et al., 2021) were correlates of the COVID-19-related organizational stress and job burnout in the remote work environment. Quality of working conditions (Mihalca et al., 2021) and perceived increased workload led to unsatisfactory professional self-esteem, psychological distress, chronic emotional and workplace stress, and high turnover. Challenges and hindrance stressors negatively affected employee wellbeing and adjustment to remote work due to the ongoing work demands (Nemţeanu and Dabija, 2021), unclear job instructions (Bernardelli et al., 2021), and work–life conflict.

Handling remote work situations inefficiently in relation to perceptions (Clark, 2020), expectations (Dobson-Lohman and Potcovaru, 2020), attitudes (Birtus and Lăzăroiu, 2021), and behaviors (Lyons and Lăzăroiu, 2020) toward organizational climate during the COVID-19 crisis (Daumiller et al., 2021; Van Zoonen et al., 2021; Venkatesh et al., 2021) hindered employee engagement and resulted in increased burnout levels. Job-related stressors were negatively associated with adjustment to remote work. Higher job demands configured employee behavior patterns (Nemţeanu and Dabija, 2020) in remote operating organizations in terms of outcomes (Morris, 2021), commitment (Bailey et al., 2021), and satisfaction. Elevated levels of work–life imbalance intensified remote employees' psychological strain.

## Conclusion

Relevant research has investigated how organizational support systems, remote work adaptation, and control over scheduling reduced psychological burnout and occupational stress (Hayes et al., 2021; Krug et al., 2021; Mahmoud et al., 2021; Van Zoonen et al., 2021), thus improving employee wellbeing during the COVID-19 pandemic. Lack of training in remote work and being new to technology, together with responsibilities determined by role in the organization and working long hours, generated increased perceived stress and precarious work–life balance. Continuity of social identity improved job satisfaction and decreased feelings of loneliness at work. Negative attitudes and behaviors and low professional motivation and engagement influenced remote workplace practices in terms of diminished productivity.

This systematic literature review has analyzed significant published peer-reviewed evidence in relation to how remote employees lacking constant organizational support during the COVID-19 outbreak (Kniffin et al., 2021; Shipman et al., 2021; Venkatesh et al., 2021) experienced escalated job demands, professional strain, low satisfaction and performance, and increased burnout. Job and financial security issues in a teleworking environment shaped remote worker support and engagement, organizational commitment, job satisfaction, and work–life balance across virtually operating organizations. Poor social connections and organizational commitment, increased job demands, and workplace loneliness affected employee health and wellbeing, negatively affecting job satisfaction and productivity.

The research results drawn from the inspected sources clarify that the COVID-19 remote work environment (Milch et al., 2021; Shipman et al., 2021; Van Zoonen et al., 2021) shaped the work–life balance and organizational commitment. Severe psychological symptoms and emotional stress were related to unsatisfactory organizational communication and increasing workload. Perceptions of organizational support and flexible work arrangements increased performance effectiveness.

## Limitations, implications, and further directions of research

Perceived stress and workplace quality, increased job demands, loneliness, and work-related burnout configured remote working experiences during the COVID-19 pandemic, shaping employee work behaviors and arrangements, career satisfaction, subjective wellbeing, turnover intentions, and job control and engagement. One limitation of this study is that we inspected only research published between 2020 and 2022 and covered only the Web of Science, Scopus, and ProQuest databases. The scope of this study does not advance how taking breaks and time management in a psychologically safe environment prevented remote work burnout and increased productivity during the COVID-19 pandemic. Subsequent analyses should focus on how remote work time and stress management by use of burnout assessment tools resulted in coherent workplace behaviors and processes meeting organizational expectations and reducing emotional stress and work pressure. Future research should investigate whether COVID-19-related unmanageable workplace stress, demanding work tasks, and excessive job expectations led to long-term negative impacts on mental health and psychological wellbeing.

## Author contributions

All authors listed have made a substantial, direct, and intellectual contribution to the work and approved it for publication.
